# Napping Reverses Increased Pain Sensitivity Due to Sleep Restriction

**DOI:** 10.1371/journal.pone.0117425

**Published:** 2015-02-27

**Authors:** Brice Faraut, Damien Léger, Terkia Medkour, Alexandre Dubois, Virginie Bayon, Mounir Chennaoui, Serge Perrot

**Affiliations:** 1 Université Paris Descartes-Sorbonne Paris Cité, APHP, Hôtel Dieu de Paris, Centre du Sommeil et de la Vigilance, Equipe d’accueil 7330 VIFASOM, Paris, France; 2 Université Paris Descartes-Sorbonne Paris Cité, APHP, Hôtel Dieu de Paris, Service de Médecine Interne et Thérapeutique, Pain Center, Paris, France; 3 IRBA (Institut de Recherche Biomédicale des Armées), Unité Fatique Vigilance, Paris, France; 4 INSERM U 987, Pain Center, Boulogne, France; Tokai University, JAPAN

## Abstract

**Study Objective:**

To investigate pain sensitivity after sleep restriction and the restorative effect of napping.

**Design:**

A strictly controlled randomized crossover study with continuous polysomnography monitoring was performed.

**Setting:**

Laboratory-based study.

**Participants:**

11 healthy male volunteers.

**Interventions:**

Volunteers attended two three-day sessions: “sleep restriction” alone and “sleep restriction and nap”. Each session involved a baseline night of normal sleep, a night of sleep deprivation and a night of free recovery sleep. Participants were allowed to sleep only from 02:00 to 04:00 during the sleep deprivation night. During the “sleep restriction and nap” session, volunteers took two 30-minute naps, one in the morning and one in the afternoon.

**Measurements and Results:**

Quantitative sensory testing was performed with heat, cold and pressure, at 10:00 and 16:00, on three areas: the supraspinatus, lower back and thigh. After sleep restriction, quantitative sensory testing revealed differential changes in pain stimuli thresholds, but not in thermal threshold detection: lower back heat pain threshold decreased, pressure pain threshold increased in the supraspinatus area and no change was observed for the thigh. Napping restored responses to heat pain stimuli in the lower back and to pressure stimuli in the supraspinatus area.

**Conclusions:**

Sleep restriction induces different types of hypersensitivity to pain stimuli in different body areas, consistent with multilevel mechanisms, these changes being reversed by napping. The napping restorative effect on pain thresholds result principally from effects on pain mechanisms, since it was independent of vigilance status.

## Introduction

Reciprocal interactions between sleep disturbances (fragmentation and restriction) and pain have been reported. A population-based study showed that sleep duration influenced pain perception with less than six hours sleep per night associated with greater pain the following day [1]. A questionnaire-based study of 882 patients with chronic low back pain found that a large proportion of these patients (42%) slept for less than six hours per night [2]. A prospective study of younger subjects found that insufficient sleep (quantity or quality) at the age of 15 to 16 years was predictive of low back pain in 18- to 19-year-old girls, even after multiple adjustments [3]. The few polysomnography recordings performed on patients with chronic pain have revealed fragmented sleep patterns and short total sleep duration [4]. Actigraphy recordings showed sleep to be less effective in 15 patients with chronic low back pain than in matched healthy subjects and sleep quality to be poor in a group of 80 patients with low back pain [5, 6].

Insufficient sleep is increasingly being associated with changes in pain perception. Marked increases in skin sensitivity to painful stimuli have been reported over a period of 60 hours of prolonged wakefulness, with no change in touch sensitivity. Epidemiological and experimental studies have suggested that fragmented and curtailed sleep result from pain while sleeping, and that fragmented and curtailed sleep may affect pain perception and tolerance [1–3, 8].

Several laboratory studies have assessed the effects of one night of sleep restriction (sleeping from 25 to 50% of a standard 8-hour night) on pain in young healthy subjects. Most have shown an increase in pain intensity or a decrease in pain thresholds [9–11].

A single night with little or no sleep can be used as an experimental model of sleep deprivation, for studying in detail the biological changes induced by a lack of sleep, whilst minimizing confounding due to the biological clock. However sleep restriction (i.e. partial sleep deprivation) is probably a more relevant model than total deprivation, reflecting more accurately the shorter sleep duration and sleep fragmentation generally experienced by patients with pain.

The contribution of napping to decreasing sleep restriction-induced hyperalgesia has never been assessed by QST and sleepiness assessments. Pain perception is a state of strong physiological reactivity, potentially modulated by vigilance processes. Sleep deficit may modulate pain-inhibiting/facilitating systems, because hyperalgesic responses may be enhanced by sleep loss but attenuated by sleep recovery (through napping).

The aim of this study was to evaluate the effect of napping on pain levels after sleep restriction, to improve our understanding of the way in which the effects of sleep restriction on vigilance influence pain tolerance.

## Materials and Methods

### 1. Ethical issues

Human studies were carried out in accordance with French regulations, with the approval of the ethics committee of Hotel-Dieu Hospital (*Comité de Protection des Personnes*, Ile de France 1) and written informed consent from participants. The participants were volunteers and all received indemnities for participation. The study was carried out in accordance with the Helsinki Declaration and ICH good clinical practice guidelines.

### 2. Subjects

Eleven healthy, male non-smokers aged 25 to 32 years (mean ± SEM: 27±1.6 years) who had a body mass index between 19 and 25 and did not take regular naps participated in this experiment. All subjects were in good health and pain-free at the initial medical examination. None had depression, anxiety or emotional distress, as assessed with the Hospital Anxiety and Depression Scale [12]. None of the volunteers had sleeping problems. All slept regular nights of seven to nine hours and were of the intermediate chronotype, as indicated by sleep and chronotype questionnaires (Pittsburgh Sleep Quality Index; Epworth Sleepiness Scale, Morningness-Eveningness Questionnaire) [13–15] and polysomnography monitoring during one night of adaptation. These volunteers were recruited by advertisements for the study at the hospital and the university campus.

### 3. Experimental design (see Fig. 1)

Each volunteer underwent two three-day sessions of testing. Volunteers were randomly assigned to two groups, one beginning with the “sleep restriction” session and the other with the “sleep restriction + nap” session. During the week preceding their admission to the sleep laboratory, participants followed a week of regular sleeping-waking behavior, with eight hours of sleep per night (in bed from 00:00 to 08:00), documented by actigraphy recordings and sleep diaries. During the study, the volunteers remained in the sleep laboratory and were continuously monitored by the investigators. Continuous ambulatory polysomnography recordings were obtained throughout the study, with a portable device (Dream, Medatec, with various EEG derivations (C4/A1, C3/A2, O2/A1, O1/A2 F4/A1, F3/A2)) for monitoring sleep and wakefulness and checking the compliance of the subjects with the imposed sleeping-waking schedule. Volunteers were free to move around within the unit, carrying this ambulatory device. During the course of the experiment, the light environment and levels of physical activity were evaluated with an ambulatory actigraphy device, recording light flux and irradiance (Philips Respironics). The subjects received controlled meals of a maximum of 2500 calories/day, with balanced nutrient contents (protein, fat, carbohydrates). The intake of medication, alcohol or xanthine derivatives (coffee, tea, chocolate or cola) was prohibited throughout the study period. Controlled drinks and snacks were available until 00:00 on sleep restriction nights. Staff members remained with the volunteers during the period of sleep restriction and the volunteers were provided with films and games during the night of sleep restriction.

**Fig 1:**
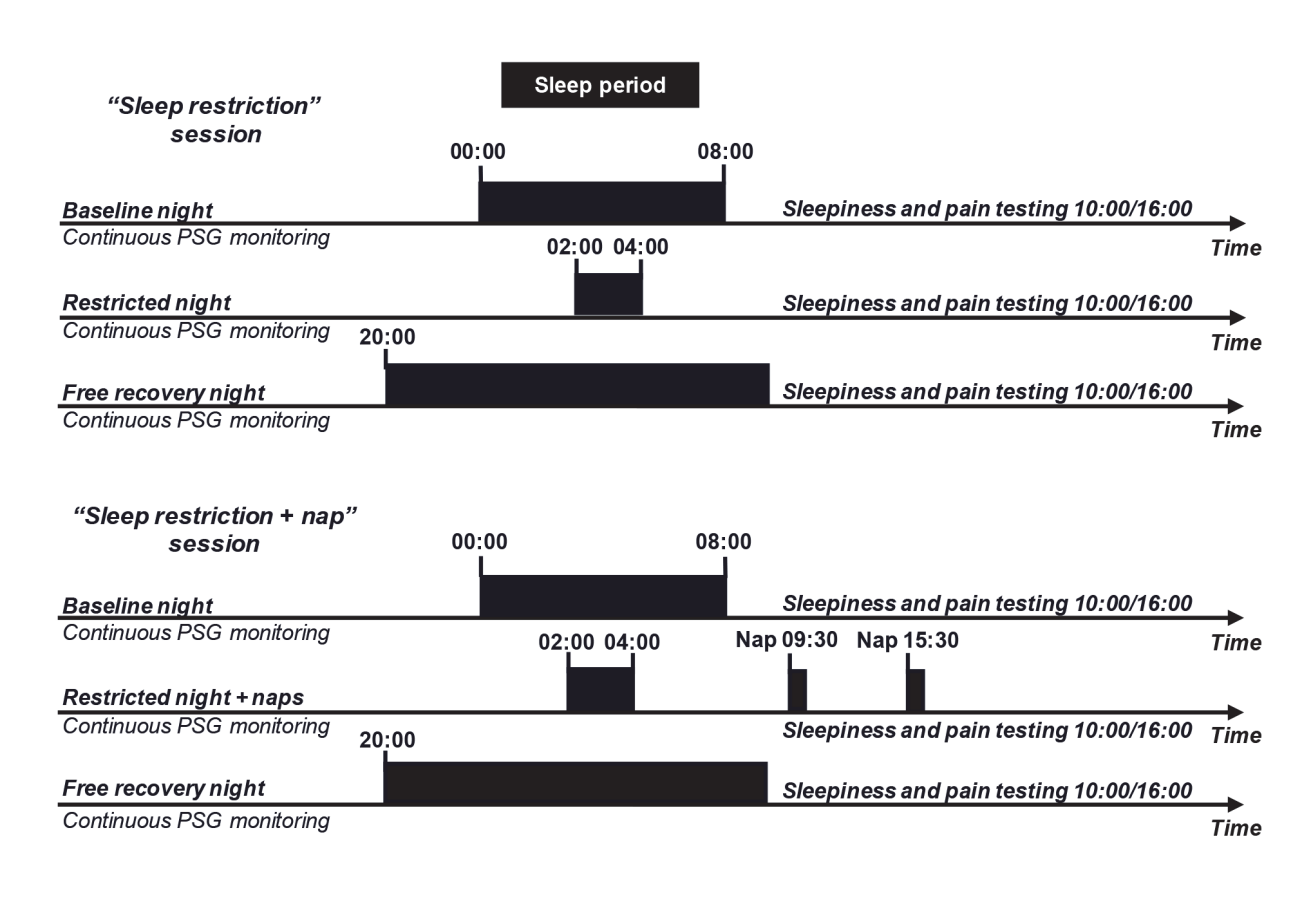
Experimental design. The experimental design included two three-day sessions, during which continuous ambulatory polysomnography (PSG) recordings were taken for each volunteer. The volunteers were randomly allocated to two groups, one of which started with the “sleep restriction” session, the other group beginning with the “sleep restriction + nap” session. In the “sleep restriction” session, the volunteers were allowed to sleep for only two hours during one night (in bed from 02:00 to 04:00), the previous night of 8 hours of sleep being used as the baseline (in bed from 00:00 to 08:00). The night after sleep deprivation, the volunteers were allowed to sleep from 20:00 until spontaneous awakening, for recovery. In the “sleep restriction + nap” session, the volunteers repeated the protocol described for the sleep restriction only session, but were allowed to take 30-minute naps at 09:30 and 15:30 on the day following the night of sleep restriction. Subjective sleepiness was assessed and QST was carried out each day, at 10:00 and 16:00.

- In the “sleep restriction” session, volunteers were allowed to sleep for only two hours in one night (in bed from 02:00 to 04:00) after a baseline night of 8 hours of sleep (in bed from 00:00 to 08:00). The following night, the volunteers were allowed to recover, by sleeping for as long as they chose, from 20:00 until spontaneous awakening the next morning.

- In the “sleep restriction + nap” session, the volunteers underwent the same protocol as for sleep restriction alone, but with two 30-minute naps, beginning at 09:30 and 15:30, on the day following the night of sleep restriction. We chose a 30-min nap since it’s a relatively short duration expected to allow about half of slow-wave sleep-the sleep stage known to be the more recuperative- as previously described with the same sleep restriction procedure [16]. We tested two times the nap effect: once in the morning and once in the afternoon at two circadian times favorable for daytime sleep [17]. Also, the ability of the subjects to perform several naps during a same day is of interest in the perspective of extended working hour conditions. For instance, interns on duty have been reported sleeping ≈ 2 hours/night on duty, a similar duration as the one we have retained in our sleep restricted protocol [18].

### 4. Sleep and sleepiness evaluation

Sleep recordings were scored visually for all subjects, according to the 2007 American Academy of Sleep Medicine criteria [19]. Subjective sleepiness was assessed on each day of each session with the Stanford Sleepiness Scale, at 10:00 and 16:00 [20]. These assessments thus took place immediately after the nap in the “Sleep restriction + nap” session

The polysomnography recordings were used to check that all subjects rigorously followed the imposed sleep-wake schedule, with no involuntary napping episodes. No significant difference in sleep architecture during the baseline and sleep-restricted nights was observed between the “sleep restriction” session and the “sleep restriction + nap” session. Most of the naps consisted of stage 2 sleep and slow-wave sleep (SWS) in roughly similar proportions (data not shown).

### 5. Quantitative sensory testing (QST)

Quantitative sensory testing (QST) was performed in a quiet room at constant temperature (22°C), by the same investigator (TM) in each case. The measurements made included the determination of thermal (heat and cold) and mechanical pain thresholds, and the responses to suprathreshold thermal stimuli. QST procedures lasted 30 minutes and were performed each day at 10:00 and 16:00, just after the evaluation of subjective sleepiness.

QST procedures were performed on three areas: the supraspinatus (on the dominant side), lower back and thigh (non-dominant side). We hypothesized that the lower back and supraspinatus areas would be sensitive to sleep restriction, whereas the thigh area would not be very sensitive to pain and could be used as a control area. This is mostly based on clinical observations, since musculoskeletal pain disorders related to sleep problems are mainly observed in neck, shoulders and back areas [21]. This is however the first sleep restriction laboratory study to investigate different sensitivities according to body area and it is therefore difficult to be sure on which area would be the more sensitive to sleep restriction. Two recent studies on spatial resolution of the pain system have demonstrated that thigh was less sensitive and discriminant to pain body area [22, 23]. For the determination of heat and cold pain thresholds, thermal sensation was assessed with a Somedic thermotest (Somedic AB, Stockholm, Sweden), by the Marstock method [24]. Briefly, a contact thermode of Peltier elements measuring 25 x 50 mm^2^ was applied to the skin. The baseline temperature of the thermode was adjusted to the patient’s skin temperature. Thresholds were measured according to the method of limits described previously [24, 25]. Stimuli of increasing or decreasing intensity were applied. For each stimulus, the subject was instructed to press a button canceling the thermal stimulation as soon as it became detectable (detection threshold), or painful (pain threshold). The interval between stimuli was 15 to 20 s for hot stimuli and 20 to 30 s for cold stimuli. The maximum and minimum temperatures were set at 50°C and 4°C. We used a rate of temperature change of 1°C/s. We also subjected the subjects to supraliminal thermal stimulation. We applied a series of suprathreshold cold and hot thermal stimuli to the skin of the subjects, as previously described [26]. Each stimulus lasted for 2 s. The intensity of the stimulus was increased to 2°C and 4°C above the pain threshold for hot stimuli and decreased to 5°C below the pain threshold for cold stimuli. After each stimulus, the subjects were asked to rate the intensity of the pain on a visual analog scale (VAS). The subjects could stop the stimulation at any time. If a VAS score of 80 or more was reported with a stimulus of a particular intensity, we did not apply stimuli of greater intensity. In such cases, we assigned the same VAS score to the higher-intensity stimulus, for the analysis of cumulative group data.

Mechanical thresholds were determined with an algometric device (Somedic). A pressure was applied to the tested area (this pressure increasing at a constant rate of 30 kPa/s) until the volunteer indicated that the pressure applied was causing pain (trains of four stimuli delivered, with an interstimulus interval of 15 seconds).

Changes in testing order can influence thresholds and ratings, and this would increase the variability of the data. We therefore carried out these tests in a fixed order: cold pain threshold, warmth detection threshold, heat pain threshold and pressure pain threshold. Thresholds were determined by calculating the arithmetic mean of three consecutive measurements.

### 6. Statistics

Data were analyzed with SigmaStat 3.5 software (Systat, San Jose, CA). Values are expressed as means (±SEM). We considered *p* < 0.05 to indicate statistical significance.

#### Within-session comparisons

The effects of sleep restriction and recovery were evaluated by two-way repeated-measures ANOVA, with a between-subject factor of sleep conditions (control, sleep restriction and free recovery), for each session separately (“sleep restriction” session and “sleep restriction + nap” session), and a within-subject factor of time (10:00, 16:00), followed by post-hoc tests for pairwise comparisons (Student—Newman—Keuls’ test).

#### Between-session comparisons

We assessed the effects of napping, by comparing the different sleep-restriction conditions (sleep restriction in the “sleep restriction” session; sleep restriction + nap in the “sleep restriction + nap” session), using normalized delta scores for each sleep restriction condition: (restriction—baseline)/baseline in the “sleep restriction” session; (restriction + nap—baseline)/baseline in the “sleep restriction + nap” session. Normalized differences with respect to baseline were then evaluated by two-way repeated-measures ANOVA, with sleep conditions as a between-subject factor (sleep restriction, sleep restriction + nap) and time as a within-subject factor (10:00, 16:00), followed by pairwise post-hoc tests (Student—Newman—Keuls’ test).

## Results

### 1. Within-session comparisons

#### 1.1 Lower back quantitative sensory testing profile

No significant difference in warmth and cold detection with respect to baseline values was observed following sleep restriction and recovery, at any of the time points tested. However, significant differences in heat pain thresholds were observed after sleep restriction (F (2, 68) = 11.1, *p* = 0.01; “sleep restriction” session, Table 1), in both the morning and the afternoon (*p* = 0.009 and 0.03, respectively, vs. control values at the same time, post hoc test). No effect of time or significant interaction between time and sleep conditions was observed (F (2, 68) = 0.04, *p* = 0.84 and F (2, 68) = 0.35, *p* = 0.57, respectively).

**Table 1:** Lower back quantitative sensory testing profile after sleep restriction without or with napping.

Area:	“Sleep restriction” session						“Sleep restriction + nap” session					
Lower back												
Sleep condition	*Control*		*Sleep*		*Recovery*		*Control*		*Sleep restriction*		*Recovery*	
			*restriction*						*+ nap*			
Test time	10:00	16:00	10:00	16:00	10:00	16:00	10:00	16:00	10:00	16:00	10:00	16:00
**Warm detection threshold (°C)**	34.8	34.8	34.8	35	35	34.6	34.3	34.6	34.4	34.7	34.5	35.9
	±1.4	±1.3	±1.5	±1.4	±1.1	±1.4	±0.9	±1.2	±0.9	±1.1	±1.1	±2.2
**Heat pain threshold**	44.1	44.1	42.6*	42.7*	43.9	44.6	43	43	42.8	42.5	43,8	43,4
**(°C)**	±0.9	±1.9	**±0.9**	**±1.2**	±1.8	±1.6	±0.9	±1.4	±0.6	±1	±1.8	±1.7
**Cold detection threshold**	29.4	29.1	29.1	29.2	28.9	29.2	29.7	29.2	29.5	29.5	29.5	29.6
**(°C)**	±0.7	±0.7	±0.6	±1.1	±0.9	±0.8	±1.3	±1.0	±1.4	±1.2	±1.3	±1.2
**Cold pain threshold**	5.5	5.9	5.3	5.6	5.4	5.1	6.8	5.2	5.6	6.3	5.1	5.8
**(°C)**	±1.1	±1.6	±0.5	±1.0	±0.7	±0.2	±3.3	±0.4	±1.1	±2.4	±0.2	±1.4
**Pressure pain**	1369	1373	1349	1337	1210	1239	1284	1290	1173	1186	1228	1307
**threshold (kPa)**	±434	±397	±372	±378	±336	±452	±421	±434	±413	±351	±366	±375

Quantitative sensory testing was performed twice daily, at 10:00 and 16:00, on the lower back area. Warmth and cold detection thresholds, and the thresholds for heat, cold and pressure pain were assessed in control, post-sleep restriction and recovery conditions in the “sleep restriction” and “sleep restriction + nap” sessions for each subjects.

Mean ± SEM;

* significant vs. corresponding control values obtained at the same time of day.

Surprisingly, when volunteers were allowed a nap before heat pain testing, no significant differences in heat pain threshold with respect to baseline values were observed on the day after sleep restriction, in either the morning or the afternoon (F (2, 68) = 1.75, *p* = 0.21; “sleep restriction + nap” session, Table 1). The decrease in heat pain thresholds induced by sleep restriction was abolished by napping, in both the morning and the afternoon. For mechanical and cold pain assessments in the lower back area, we observed no significant difference in threshold values with respect to baseline following sleep restriction and recovery, at any of the time points tested.

#### 1.2 Supraspinatus quantitative sensory testing profile

Sleep restriction and rest had no significant effect on warmth and cold detection thresholds or heat and cold pain thresholds for the supraspinatus area, at any of the time points tested (Table 2). Supraliminal thermal stimulation scores in response to heat or cold stimuli were unaffected by sleep conditions (Table 3). By contrast, pressure pain threshold was significantly decreased by sleep restriction (F (2, 68) = 6.232, *p* = 0.03; “sleep restriction” session, Table 2) at both 10:00 and 16:00 (*p* = 0.04 and 0.03, respectively, for comparisons with the same-time baseline, post hoc test). No effect of time or significant interaction between time and sleep conditions was observed (F (2, 68) = 0. 0566, *p* = 0.818 and F (2, 68) = 0.130, *p* = 0.727, respectively). In the “sleep restriction + nap” session, pressure pain thresholds did not differ from baseline values after the morning and afternoon naps (F (2, 68) = 0.52, *p* = 0.48; “sleep restriction + nap” session, Table 2). Napping restored mechanical pain thresholds to baseline levels, both in the morning and in the afternoon.

**Table 2:** Supraspinatus quantitative sensory testing profile after sleep restriction without or with napping.

Area:	“Sleep restriction” session						“Sleep restriction + nap” session					
Supraspinatus												
Sleep condition	*Control*		*Sleep*		*Recovery*		*Control*		*Sleep restriction*		*Recovery*	
			*restriction*						*+ nap*				
Test time	10:00	16:00	10:00	16:00	10:00	16:00	10:00	16:00	10:00	16:00	10:00	16:00
**Warm detection threshold (°C)**	35.6	35.7	35.9	35.4	36.1	35.8	34.7	34.9	35.2	35.8	35.5	36.8
	±1.7	±1.8	±1.4	±0.8	±1.0	±1.8	±0.9	±1.0	±1.0	±1.9	±1.3	±2.7
**Heat pain threshold**	46.1	45.9	45.7	45.8	46.4	46.1	45.1	45.5	45.1	45.2	45.7	45.6
**(°C)**	±2.3	±1.6	±2.6	±2.2	±1.8	±2.1	±2.4	±1.6	±1.8	±1.5	±1.7	±2.0
**Cold detection threshold**	29.5	29.4	29.5	29.4	29.1	29	29.2	29.4	29.2	28.4	28.9	29.4
**(°C)**	±1.1	±1.0	±0.9	±1.0	±1.3	±0.8	±1.0	±1.3	±1.8	±2.7	±1.9	±1.3
**Cold pain threshold**	5.6	6	5.4	5.6	5.1	5.2	5.5	5.2	5.3	6.2	5.1	5.2
**(°C)**	±1.0	±1.8	±0.8	±1.1	±0.2	±0.3	±0.9	±0.4	±0.4	±2.1	±0.2	±0.3
**Pressure pain**	1209	1238	973*	1065*	1109	1133	1104	1048	974	936	1029	948
**threshold**	±328	±306	**±177**	**±227**	±195	±126	±212	±289	±247	±207	±244	±164
**(kPa)**												

Quantitative sensory testing was performed twice daily, at 10:00 and 16:00, on the supraspinatus area. Warmth and cold detection thresholds, and the thresholds for heat, cold and pressure pain were assessed in control, post-sleep restriction and recovery conditions, in the “sleep restriction” and “sleep restriction + nap” sessions for each subject.

Mean ± SEM;

* significant vs. the corresponding control values obtained at the same time of day.

**Table 3:** Supraliminal thermal stimulation in supraspinatus area after sleep restriction without or with napping.

Area:	“Sleep restriction” session						“Sleep restriction + nap” session					
Supraspinatus												
Sleep condition	*Control*		*Sleep*		*Recovery*		*Control*		*Sleep restriction*		*Recovery*	
			*restriction*						*+ nap*			
Test time	10:00	16:00	10:00	16:00	10:00	16:00	10:00	16:00	10:00	16:00	10:00	16:00
												
**Pain ratings**												
**(mm VAS)**												
**40°C**	1.6	5.1	3.5	4.8	1.4	3	7.5	3.4	4	5.4	3.2	2.4
	±2.9	±8.1	±5.6	±7.6	±2.5	±4.8	±9.6	±5.6	±6.5	±6.9	±5.4	±3.9
**44°C**												
	30.1	34.4	30.6	27.4	29.4	30.4	40.1	33.5	36.7	31.7	27.6	27.3
	±25.3	±19.7	±22.1	±21.2	±23.5	±22.2	± 19.1	±13.5	±18.9	±23.5	±22.9	±17.7
**48°C**												
	40.5	47.1	47.2	48.6	42.2	39.6	50.1	48	49.4	48	41.2	45.8
	±16.5	±11.5	±10.8	±20.2	±16.6	±20.7	±17.0	±14.9	±10.7	±12.5	±15.5	±10.7
**15°C**	0	0	0	0	0	0	0	0	0	0	0	0
	±0.0	±0.0	±0.0	±0.0	±0.0	±0.0	±0.0	±0.0	±0.0	±0.0	±0.0	±0.0
**10°C**												
	2.1	0	2.1	2	1.5	2.3	5.2	0	0	0	1.4	0
	±3.7	±0.0	±3.8	±3.6	±2.7	±4.1	±8.5	±0.0	±0.0	±0.0	±2.5	±0.0
**5°C**												
	5.6	3.4	5.1	4.7	5.9	4.8	11	4.9	4	2.6	4.5	2.7
	±8.5	±5.4	±9.1	±8.4	±9.4	±8.4	±14.0	±7.8	±6.5	±4.8	±7.4	±4.4

Quantitative sensory testing was performed twice daily, at 10:00 and 16:00, on the supraspinatus area. Supraliminal hot and cold thermal stimuli were applied in control, post-sleep restriction and recovery conditions, in the “sleep restriction” and “sleep restriction + nap” sessions for each subject. Following each stimulus, subjects were asked to rate the intensity of the pain on a VAS.

Mean ± SEM.

#### 1.3 Thigh quantitative sensory testing profile

We hypothesized that the thigh area could be used as a control area, insensitive or less sensitive to the effects of a loss of sleep. Consistent with this hypothesis, we observed no change in tolerance to cold, heat or pressure pain the day following the sleep restricted night. Heat pain threshold in the morning session after the recovery night of sleep was found to be higher than that in control conditions (F (2, 68) = 9.90, *p* = 0.01; *p* = 0.04 vs. control 10:00, post hoc test; “sleep restriction + nap” session, Table 4).

**Table 4:** Thigh quantitative sensory testing profile after sleep restriction without or with napping.

Area:	“Sleep restriction” session						“Sleep restriction + nap” session					
Thigh												
Sleep condition	*Control*		*Sleep*		*Recovery*		*Control*		*Sleep restriction*		*Recovery*	
			*restriction*						*+ nap*			
Test time	10:00	16:00	10:00	16:00	10:00	16:00	10:00	16:00	10:00	16:00	10:00	16:00
**Warm detection threshold (°C)**	36.3	36.3	36.1	36.7	35.7	36.6	35.2	35.7	36.1	37.9	36.2	37.3
	±1.8	±0.9	±1.3	±1.0	±1.8	±1.5	±0.9	±0.8	±1.3	± 2.8	±0.9	±2.8
**Heat pain threshold**	46.1	46.1	46.2	45.3	46.8	46.8	45.6	46	46.1	46	47.0*	47.1
**(°C)**	±1.0	±1.5	±1.3	±2.2	±2.2	±1.8	±1.7	±1.4	±1.9	±1.4	**±1.8**	±1.9
**Cold detection threshold**	28.8	28.6	29	28.5	29.5	28.7	28.8	28.7	28.2	28.5	28	30.3
**(°C)**	±1.1	±1.2	±1.5	±1.1	±2.4	±1.1	±1.7	±2.0	±2.1	±2.1	±2.6	±3.7
**Cold pain threshold**	5.2	5.9	5.3	5.2	5.2	5.1	5.8	5.2	5.7	5.6	5.1	5.1
**(°C)**	±0.3	±1.6	±0.6	±0.3	±0.2	±0.2	±1.5	±0.5	±1.2	±1.0	±0.2	±0.3
**Pressure pain**	1570	1728	1656	1643	1661	1659	1599	1559	1558	1479	1592	1618
**threshold**	±274	±452	±371	±372	±462	±455	±472	±602	±496	±494	±602	±530
**(kPa)**												

Quantitative sensory testing was performed twice daily, at 10:00 and 16:00, on the thigh area. Warmth and cold detection thresholds, and the thresholds for heat, cold and pressure pain were assessed in control, post-sleep restriction and recovery conditions, in the “sleep restriction” and “sleep restriction + nap” sessions, for each subject.

Mean ± SEM;

* significant vs. the corresponding control values obtained at the same time of day.

#### 1.4 Stanford sleepiness scale

Significant differences in Stanford scale ratings were obtained on the day after sleep restriction (F (2, 68) = 32.51, *p* < 0.001; “sleep restriction” session, Table 5). Sleepiness levels at 10:00 and 16:00 after sleep restriction were almost double the control values for the same time points (*p* = 0.002 and 0.003, respectively, vs. the same-time control value, post hoc test). No effect of time or significant interaction between time and sleep conditions was detected (F (2, 68) = 1.73; *p* = 0.19 and F (2, 68) = 0.07; *p* = 0.97, respectively). Similar changes were observed in the “sleep restriction + nap” session (F (1, 45) = 55.06 *p* < 0.001), Sleepiness after napping was greater than that in control conditions, at 10:00 and 16:00 (both *p* < 0.001 vs. same-time control values, post hoc test). No effect of time or significant interaction between time and sleep conditions was detected (F (2, 68) = 0.15, *p* = 0.70 and F (2, 68) = 0.78, *p* = 0.39, respectively). No significant differences in sleepiness with respect to control values were observed following the recovery night.

**Table 5:** Stanford sleepiness scale after sleep restriction without or with napping.

	“Sleep restriction” session						“Sleep restriction + nap” session					
Sleep condition	*Control*		*Sleep*		*Recovery*		*Control*		*Sleep restriction*		*Recovery*	
			*restriction*						*+ nap*			
Test time	10:00	16:00	10:00	16:00	10:00	16:00	10:00	16:00	10:00	16:00	10:00	16:00
**Stanford sleepiness scale**	1.5	1.7	2.8*	3.1*	1.7	1.5	1.6	1.6	3.2*	3.5*	1.3	1.2
	±0.5	±0.5	**±0.9**	**±0.9**	±0.7	±0.7	±0.6	±0.7	**±0.7**	**±0.6**	±0.5	±0.3

Subjective sleepiness was tested on each day of each session, at 10:00 and 16:00. Stanford Sleepiness Scale scores are shown for control, post-sleep restriction and recovery conditions, for the “sleep restriction” and “sleep restriction + nap” sessions.

Mean ± SEM;

* significant vs. the corresponding control value obtained at the same time of day.

### 2. Between-session comparisons

**Fig 2:**
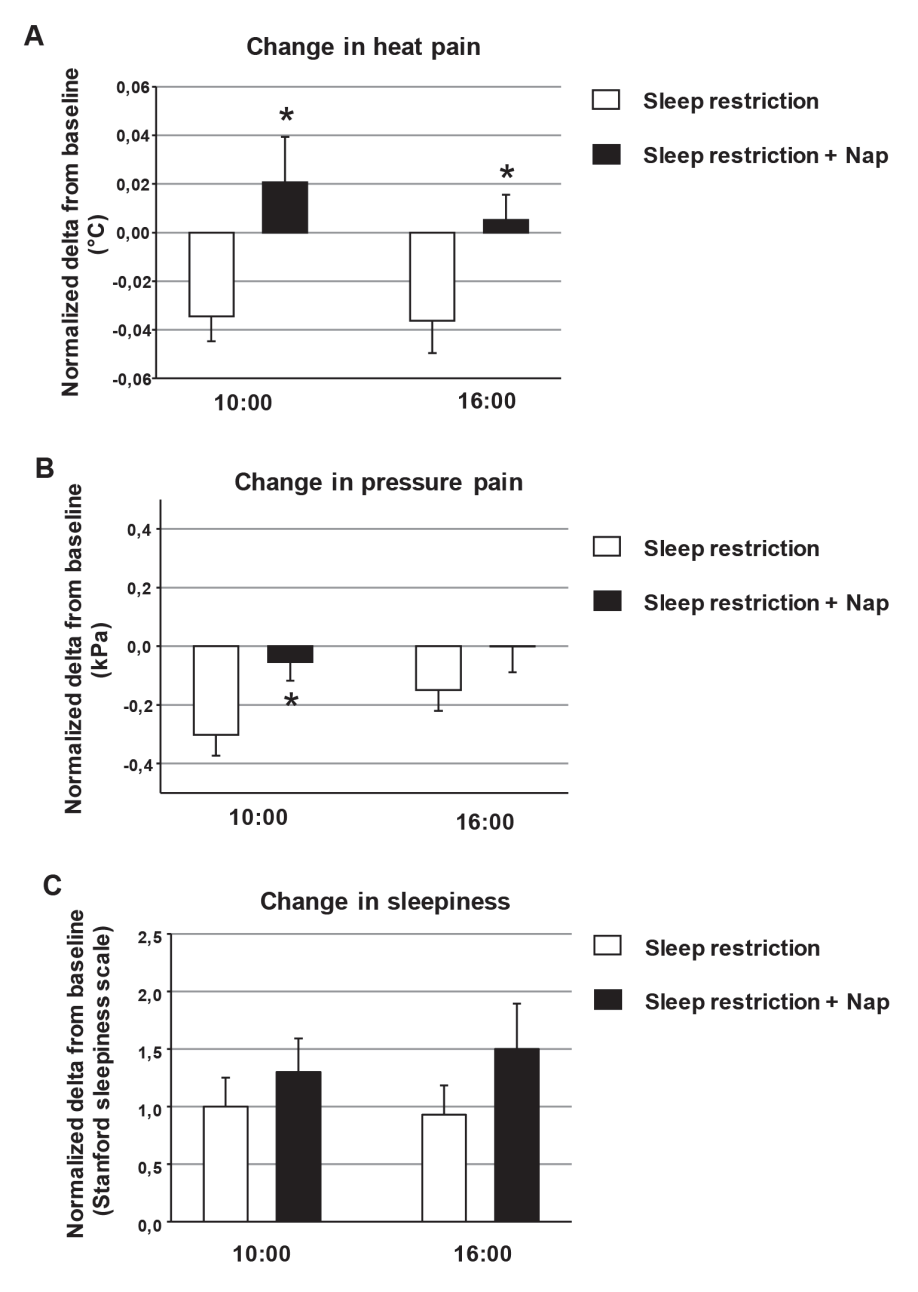
Changes in heat pain, pressure pain and sleepiness without or with napping after sleep restriction. We assessed the effects of napping, by comparing the different sleep restriction conditions (“sleep restriction” session and “sleep restriction + nap” session), using normalized delta scores for each sleep restriction condition relative to the corresponding baseline. Changes are shown for heat pain (A), pressure pain (B) and sleepiness (C). Sleep restriction increased sleepiness and decreased pain tolerance, whereas napping reversed the increase in pain sensitivity but did not decrease sleepiness (due to sleep inertia). Mean ± SEM; * indicates significant differences between conditions.

We assessed the effects of napping, by comparing normalized changes from baseline between the “sleep restriction” and “sleep restriction + nap” sessions (Fig. 2).

In the lower back area, the effects of sleep restriction on heat pain thresholds varied depended on the sleep restriction conditions (F (1, 45) = 20.3, *p* = 0.002). Pairwise between-session comparisons showed a significant difference between the “sleep restriction” and ‘‘sleep restriction + nap” sessions, indicating an analgesic effect of napping, at both the 10:00 and 16:00 time points (*p* = 0.015 and 0.039, respectively, post hoc test; Fig. 2A).

The effects of sleep loss on mechanical pain thresholds in the supraspinatus area depended on the sleep restriction conditions (F (1, 45) = 8.8, *p* = 0.01). The morning nap counteracted the increase in sensitivity to pressure pain more effectively than the afternoon nap (*p* = 0.005 and 0.65 respectively, post hoc test; Fig. 2B).

By contrast, the effects of sleep restriction on the levels of sleepiness at 10:00 and 16:00 were not affected by napping (F (1,41) = 0.71; *p* = 0.42; Fig. 2C). The increase in sleepiness relative to baseline was therefore similar after sleep restriction alone and after sleep restriction with morning and afternoon naps. The changes to heat pain thresholds for the lower back and pressure pain thresholds in the supraspinatus area were independent of sleepiness levels.

## Discussion

### 1. Main findings

The major findings of this study were as follows: i) sleep restriction (only 2 hours of nocturnal sleep) induces significant changes in heat and mechanical pain thresholds, which differ as a function of the area tested, without altering the detection thresholds for warmth and cold, ii) for both mechanical and heat stimuli, hyperalgesia was abolished by a 30-minute nap in the morning or the afternoon iii) the restoration of normal pain threshold by napping was independent of vigilance status. We also noted a time-dependent/circadian effect of napping on pressure pain, with morning naps having greater analgesic efficacy than afternoon naps.

### 2. Differential effects of sleep restriction as a function of the type of pain stimulus

#### Cold hyperalgesia

Cold hyperalgesia was not observed in any of the volunteers, at baseline, after sleep restriction or after a nap. This finding differs from those of Schuh-Hofer *et al*. [27] who reported cold hyperalgesia as a prominent finding, but are consistent with those of Kundermann *et al*. [28] who reported only a non-significant trend towards a decrease in cold pain threshold after sleep restriction. These differences may be related to the area stimulated (see below). In the Schuh—Hofer study [27], the hand was stimulated. This distal area of the body is probably more sensitive to cold and to vascular changes than the areas analyzed in our study. A recent study reporting hypersensitivity to cold pain after sleep deprivation suggested that cold stimuli may induce local vasomotor changes [29]. The vascular modifications induced by thermal stimuli in distal parts of the body, such as the hand, could be assessed by Döppler analyses in QST studies.

#### Heat hyperalgesia

Sleep restriction led to a significant decrease in heat pain thresholds in the lower back area. This finding is consistent with those of previous studies [9, 11, 27, 28]. Sleep deprivation has already been shown to decrease the time to finger withdrawal in response to a heat stimulus, this decrease being greater in the afternoon than in the morning (comparisons made to results obtained in control conditions, at the same time of day), but napping was not evaluated [10].

#### Mechanical hyperalgesia

Sleep restriction also resulted in an increase in sensitivity to pain caused by mechanical pressure. This finding is consistent with many reports of mechanical (pressure) hyperalgesia after sleep restriction, particularly in patients with fibromyalgia [9, 30, 31].

The normalized pressure pain thresholds changes from baseline within the “sleep restriction” session were significantly higher the morning than the afternoon and displayed similarly to nap efficiency—more efficient the morning- a time-dependent effect. This suggests that these differences for threshold mechanical pain changes (higher in the morning) results from a circadian drive effect rather from the homeostatic sleep pressure, expected inversely to be higher in the afternoon.

### 3. Differential effects of pain stimuli as a function of the anatomic area tested

Pain thresholds were recorded in three areas: two areas frequently implicated in sleep deprivation-related pain (the trapezius on the dominant side and the lower back), and a control area (the contralateral thigh). Our results indicated that the lower back and trapezius were affected differently by different types of nociceptive stimulation. Following sleep deprivation, the lower back area was found to be more sensitive to thermal pain stimuli than before sleep deprivation, whereas no significant change was reported for the supraspinatus area. Conversely, the supraspinatus area displayed a decrease in threshold to pressure pain, whereas the threshold of the lower back to such pain was unaffected. These differences may reflect differences in local peripheral innervation and in vasomotor reactions to thermal stimuli. The lower back area frequently receives mechanical inputs and may, therefore, be less sensitive to mechanical stimuli.

### 4. Spontaneous pain

Consistent with the findings of previous studies [27, 28], none of the participants complained of spontaneous pain after sleep restriction. Most previous studies have suggested that an accumulation of sleep restriction over time is required for healthy volunteers to begin to complain of pain [32].

### 5. Effect of napping on changes in pain thresholds

This is the first study, to our knowledge, to demonstrate that short napping episodes can restore baseline pain sensitivity in subjects with pain hypersensitivity induced by sleep restriction. A previous study demonstrated that sleep extension could reverse pain hypersensitivity related to a chronic mild loss of sleep.^10^ Studies in animals have also demonstrated significant changes in pain thresholds after sleep deprivation, with a sustainable effect lasting 96 hours [33] and the restoration of normal pain thresholds after only six days of sleep recovery [34].

### 6. Limitations of the study

We acknowledge that this study was performed in a relatively small sample size of subjects although their healthy status is expected to reduce inter-subject variability. In addition, the relatively small sample size of subject could be a potential contributing factor to some inter-session mean variability observed in control condition for heat and pressure pain thresholds. Although the control conditions were not significantly different between each session, a higher number of subjects included would have probably blunted this variability and strengthened our data. Also, several additional days of sleep restriction might have probably consolidated sleep loss effects and reduced the body area heterogeneity response to pain stimuli measured after one night of sleep restriction.

Finally, the morning-after the recovery night preceded by the nap sessions but not for sleep recovery without nap- was associated with lower levels of heat pain nociception in the thigh area. This suggests that although no changes were measured after sleep restriction in any QST pain parameters, the thigh area could be not as “immune” to the degree of sleep recovery regarding heat pain tolerance.

All the experiments described here were then performed in healthy volunteers, but it would be of interest to perform the same tests in patients with chronic pain (e.g. low back pain, shoulder pain and diffuse pain) and/or for several consecutive nights of sleep restriction. Our findings also suggest that QST protocols should not be unique, and that these protocols should be adapted to the conditions and the aims of the study.

### 7. Effects of sleep restriction on pain: changes in perception mediated by a decrease in alertness?

Sleep deprivation has well known adverse effects on cognition and vigilance [35]. The results obtained may have been biased by an impairment of the level of vigilance in the subjects. However, the change in heat pain thresholds but not in thermal detection thresholds suggests that this was not the case. Furthermore, napping restored normal pain thresholds but had no effect on sleepiness.

It has been suggested that the perceived intensity of nociceptive stimuli depends on vigilance state. Indeed, pain nociception, as assessed by the time to finger withdrawal following exposure to a radiant heat stimulus, has been shown to be specifically associated with an increase in alertness in healthy subjects [10, 36]. One group compared pain nociception before and after a night of extended sleep, in sleepy individuals [10]. Another group compared a non-sleepy group of healthy subjects (as defined by a latency > 8 min at the multiple sleep latency test) to a group of sleepier subjects (as defined by a latency < 8 min at the multiple sleep latency test) [36]. Increases in objective sleepiness were found to be associated with decreases in pain threshold. The extremely low sleepiness score following the recovery night for the “sleep restriction + nap” session was associated with significantly lower levels of heat pain nociception in the thigh area than after a normal eight-hour night of sleep. Despite differences in the methods used to measure pain and sleepiness between these two studies, the extended night of sleep for sleepy individuals in the previous study appears to be comparable to the recovery night of sleep in our study, which resulted in 54.5 ± 6.7% of extra total sleep time respect to the baseline night.

However, the effects of sleep inertia suggest that the links between the state of vigilance of the subject and pain perception are more complex. Sleep inertia is a transitional period of diminished arousal after awakening, characterized by poor cognitive task performance and a sensation of disorientation, which is usually worse if the subject is awoken during SWS and usually dissipates within 30 minutes of waking from a nap [37–39]. Many previous studies have assessed the effects of sleep inertia on psychomotor vigilance or higher cognitive decision-making tasks, fewer investigations have focused on the effects of sleep inertia on pain modulation and perception. In our study, the sleep-deprived subjects taking part in the “sleep deprivation + nap” session had the opportunity to take a 30-minute nap, about half of which consisted of SWS (the proportion of SWS was higher for the afternoon nap than for the morning nap). The determination of Stanford sleepiness ratings within a few minutes of waking after the 30-minute nap showed a negative effect of sleep inertia, with a lack of decrease in sleepiness immediately after the nap. However, improvements in Stanford sleepiness scores were noted some time later, at 19:00, for subjects taking naps, whereas no such improvement was observed in the absence of napping (data not shown). Thus, sleep restriction increases sleepiness and decreases pain threshold, whereas napping also leads to an increase in sleepiness (due to sleep inertia) but reverses the changes in pain tolerance triggered by sleep deprivation. In our study we did not use objective assessment of reaction time and lapses, like the Psychomotor Vigilance Test (PVT) [40]. It may be usefully tested in future studies in order to better understand the implication of attention in pain.

### 8. Conclusions

Poor sleep and pain are two crucial fields of public health. Many recent studies have reported that between 20 and 30% of adults currently sleep less than 6 hours per 24-hour period, resulting in a growing sleep deficit [41, 42]. While some data reported hyperalgesic changes using QST after one night of total sleep deprivation, our data indicated that one night of partial sleep deprivation, i.e. acute sleep restriction was sufficient to elicit most of these hyperalgesic responses. Sleep restriction and even more chronic sleep restriction are more relevant than total sleep deprivation in the context of sleep-deprived workers during the week and pain patients. Our results suggest that the fragmented, curtailed sleep frequently experienced by patients with chronic pain would be expected to intensify pain symptoms in a vicious circle of hypersensitivity to various pain stimuli. Napping has been reported to improve alertness, concentration, mood and immunity [16, 43–45]. Our findings also suggest that napping may reduce pain hypersensitivity, regardless of vigilance status. Future studies should investigate how napping could be used to decrease analgesic intake in patients with chronic pain.
